# Immunomodulatory Effects of Kuseonwangdogo-Based Mixed Herbal Formula Extracts on a Cyclophosphamide-Induced Immunosuppression Mouse Model

**DOI:** 10.1155/2018/6017412

**Published:** 2018-04-08

**Authors:** Joo Wan Kim, Jae-Suk Choi, Du Jin Seol, Jai Jun Choung, Sae Kwang Ku

**Affiliations:** ^1^Aribio Co. Ltd., No. 2-301, Pangyo Seven Venture Valley, 15 Pangyoro 229-gil, Bundang-gu, Sungnam, Gyeonggi-do 13487, Republic of Korea; ^2^Division of Bioindustry, College of Medical and Life Sciences, Silla University, 140 Baegyang-daero 700 Beon-gil, Sasang-gu, Busan 46958, Republic of Korea; ^3^Department of Anatomy and Histology, College of Korean Medicine, Daegu Haany University, Hanuidae-ro, Gyeongsan-si, Gyeongsangbuk-do 38610, Republic of Korea

## Abstract

**Aim:**

Kuseonwangdogo is a traditional Korean immunomodulatory polyherbal prescription. However, there are no systemic findings on its complex immunomodulatory effects on* in vivo* models. In this study, we observed the immunomodulatory effects of Kuseonwangdogo-based mixed herbal formula aqueous extracts (MHFe) on cyclophosphamide- (CPA-) induced immunosuppression mouse model.

**Methods:**

In total, 60 male 6-week-old ICR mice (10 mice/group) were selected based on body weight 24 h after the second CPA treatment and used in this experiment. Twelve hours after the end of the last (fourth) oral administration of MHFe, the animals were sacrificed.

**Results:**

Following CPA treatment, a noticeable decrease in the body, thymus, spleen, and submandibular lymph node (LN) weights; white blood cell, red blood cell, platelet number, hemoglobin, and hematocrit concentrations; serum interferon-*γ* levels; splenic tumor necrosis factor-*α*, interleukin- (IL-) 1*β*, and IL-10 content; and peritoneal and splenic natural killer cell activities was observed. Depletion of lymphoid cells in the thymic cortex, splenic white pulp, and submandibular LN-related atrophic changes were also observed. However, these CPA-induced myelosuppressive signs were markedly and dose-dependently inhibited by the oral administration of 125, 250, and 500 mg/kg MHFe.

**Conclusion:**

MHFe can be a promising, potent immunomodulatory therapeutic agent for various immune disorders.

## 1. Introduction

Numerous factors can influence the immune system development, maintenance, and optimal functioning [1]. Therefore, modulation by suppressing or stimulating the immune responsiveness of an organism against the invading antigen and alleviating the disease has been of interest for many years [2, 3]. Furthermore, many of the currently available immunomodulators, such as levamisole, glucans, telerones, and L-fucose, as well as* Corynebacterium parvum* bacterium, have side effects such as fever, neutropenia, leucopenia, and allergic reactions [3, 4]. Hence, identifying better agents and evaluating their immunomodulatory potential is gaining attention globally [3]. True immunomodulation includes stimulation and suppression of the immune system [2].

Nutrition impacts physiological processes in the body and nutritional status can have important implications on immune functions, resistance to infection, and autoimmunity [5]. Certain nutrients play a crucial role in the maintenance of optimum immune responses and their deficiency or excessive intake could adversely affect the number and activity of the immune cells [3]. Nutrients support the immune system by providing antioxidants. Immune cells, such as T cells, natural killer (NK) cells, and T-helper cells, are characterized by excessive levels of reactive oxygen species (ROS), which are employed, in part, to kill ingested pathogens. In addition, immune cell membranes are enriched with polyunsaturated fatty acids susceptible to ROS-mediated damage [3, 6]. Therefore, supplementation using nutrients with antioxidant properties, such as carotenes, vitamin E, vitamin C, zinc, selenium, and polyphenols, may quench these free radicals and influence several components of the immune system [3, 7, 8]. Natural herbs contain various phenolic compounds, vitamins, carotenoids, and flavonoids and have various pharmacological effects including immunomodulatory, antioxidative, antiallergic, and anticancer effects [9, 10]. Therefore, there has been a growing interest in the field of herbal medicines and search for promising potential compounds for investigating immunomodulatory compounds from natural products in recent years [11]. Herbal drugs enhance the natural resistance of the body against infection and numerous plants have immunomodulatory activities [12, 13].

Kuseonwangdogo is a traditional Korean immunomodulatory polyherbal prescription [14]. It comprises seven herbs [Nelumbinis Semen (150 g), Dioscoreae Rhizoma (150 g), Hoelen Alba (150 g), Coicis Semen (150 g), Hordei Fructus Germinatus (75 g), Lablab Semen Album (75 g), and Euryales Semen (75 g)] and two sweeteners [Korean traditional sweetener, Shi-sang, prepared from dried persimmon (37.5 g) and sugar (750 g)]. Ju et al. (1999) [14] reported the* in vitro* anticomplementary effects of Kuseonwangdogo. Jung et al. (1996) [15] suggested that Kuseonwangdogo extracts may have immunostimulatory effects. In addition, all the seven herbal components of Kuseonwangdogo, Nelumbinis Semen [16], Dioscoreae Rhizoma [17], Hoelen Alba [18], Coicis Semen [19], Hordei Fructus Germinatus [20], Lablab Semen Album [21], and Euryales Semen [22], have been shown to have direct immunomodulatory effects or related antioxidant effects. However, there are no systemic findings on the complex immunomodulatory effects of Kuseonwangdogo extracts. In particular, CPA-induced immunosuppression mouse model has been used as valuable animal model for detecting antimutagenic or favorable immunomodulatory effects [23–25].

In this study, the immunomodulatory effects of the two sweeteners in Kuseonwangdogo-based mixed herbal formula aqueous extracts (MHFe; yield = 11.03%; Table 1) on CPA-induced immunosuppression mouse model were observed. To induce immunosuppression in mice, CPA was intraperitoneally injected twice 3 days or 1 day before the initial test substance administration at dosages of 150 and 110 mg/kg (of body weights). Test substances were orally administered 4 times 24 h after second CPA treatment at 12 h intervals according to our previously established method [25]. *β*-glucan, a well-documented immunomodulatory polysaccharide, at 250 mg/kg was used as the reference drug according to previous studies [25–28]. Twelve hours after the last (fourth) oral administration of 125, 250, and 500 mg/kg MHFe or 250 mg/kg *β*-glucan, changes in body, thymus, spleen, and submandibular lymph node (LN) weights, 13 hematological parameters (Table 2), serum interferon- (IFN-) *γ* levels, peritoneal and splenic natural killer (NK) cell activities, and splenic tumor necrosis factor- (TNF-) *α*, interleukin- (IL-) 1*β*, and IL-10 levels were monitored with histopathology of lymphoid organs. The total and cortex thicknesses of thymus, white pulp numbers, total and white pulp diameters of the spleen, lymphoid follicle numbers, total and cortex thicknesses of submandibular LN were the histomorphometrical parameters investigated in this study.

## 2. Materials and Methods

### 2.1. Animals

In total, 60 healthy male SPF ICR mice (6 weeks old upon receipt; Orient Bio, Seongnam, Korea; body weight 28–32 g upon receipt) were used after acclimatization for 7 days. Four animals were allocated per polycarbonate cage kept in a temperature (20–25°C) and humidity (50–55%) controlled room with 12 h light/dark cycle and fed standard rodent chow (Samyang, Seoul, Korea); water was available ad libitum. All laboratory animals were treated according to the international regulations for the usage and welfare of laboratory animals and the study protocol was approved by the Institutional Animal Care and Use Committee in Daegu Haany University (Gyeongsan, Gyeongbuk, Korea) [Approval Number DHU2014-068] before animal experiment. Six groups, 10 mice in each group, were selected based on the body weight deviations at 24 h after second CPA treatment (total 50 immunosuppressive mice, average 34.67 ± 1.64 g; 10 intact mice, average 37.81 ± 2.14 g) and used in this experiment as follows:

*Experimental Groups (Eight Mice per Group Were Finally Sacrificed)*

1. Vehicle control: distilled water-administered intact mice
2. CPA control: CPA-treated and distilled water-administered control mice
3. *β*-glucan: CPA-treated and 250 mg/kg *β*-glucan administered mice
4. MHFe 500: CPA-treated and 500 mg/kg MHFe administered mice
5. MHFe 250: CPA-treated and 250 mg/kg MHFe administered mice
6. MHFe 125: CPA-treated and 125 mg/kg MHFe administered mice.

### 2.2. Preparation and Administration of Test Materials

The two-sweetener-excluded Kuseonwangdogo-based MHFe (brown solution) were prepared from the appropriated mixtures (4.785 kg) of the seven herbs, as listed in Table 1 [Nelumbinis Semen (870 g), Dioscoreae Rhizoma (870 g), Hoelen Alba (870 g), Coicis Semen (870 g), Hordei Fructus Germinatus (435 g), Lablab Semen Album (435 g), and Euryales Semen (434 g)], in 40 L of distilled water. The extracts were stored at room temperature for 8 h. Then, they were boiled at 90°C for 13 h and filtered (200 mesh). Concentrated solutions (30.580 L) of 1.1°Brix were obtained. Concentrated solutions, as 1.1 brix, were acquired. MHFe powder was prepared from this solution (yield 11.03%, total weight 527.7 g) using a rotary vacuum evaporator (N-1110, Eyela, Tokyo, Japan) and programmable freeze dryer (FDB-5503, Operon, Kimpo, Korea) and used in this experiment as test materials. Specimens of lyophilized aqueous extracts of MHFe were deposited in the herbarium of the Medical Research Center for Globalization of Herbal Formulation, Daegu Haany University (Code KSWDG2014Ku01). Light brown powder of *β*-1,3/1,6-glucan purified from* Aureobasidium pullulans* SM2001 (Glucan Corp., Busan, Korea) was used as the reference drug. MHFe and *β*-glucan were stored in a refrigerator at −20°C and 4°C, respectively, until use. MHFe solution (50 mg/ml) in distilled water and *β*-glucan solution (25 mg/ml) in distilled water were used in this experiment.

**Figure 1:**
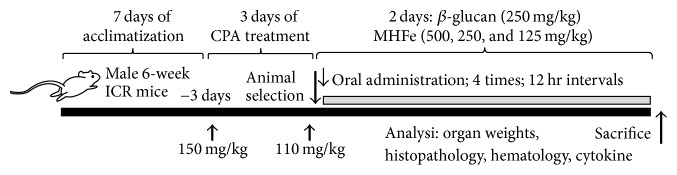
Experimental designs used in this study. CPA: cyclophosphamide; MHFe: two-sweetener-excluded Kuseonwangdogo-based mixed herbal formula aqueous extracts.

The dosage of *β*-glucan was selected as 250 mg/kg based on previous* in vivo* efficacy tests [25–28]. The middle dosage of MHFe was selected as 250 mg/kg for direct comparison with *β*-glucan, and 500 and 125 mg/kg were selected as the highest and lowest dosages using common ratio 2, respectively. Appropriated MHFe and *β*-glucan were dissolved in distilled water and orally administered at 10 ml/kg 4 times at 12 h intervals 24 h after second CPA treatment. In intact and CPA control mice, 10 ml/kg of distilled water was orally administered instead of test substances according to our previously established method [25] (Table 2, Figure 1).

### 2.3. CPA-Induced Immunosuppression

CPA, dissolved in sterilized saline (10 ml/kg), was twice intraperitoneally injected 3 days or 1 day before initial test substance administration at dosages of 150 and 110 mg/kg to induce immunosuppression in mice after 7 days of acclimatization according to our previous established method [25]. In intact control mice, an equal volume of sterilized saline was intraperitoneally injected (Table 2, Figure 1).

### 2.4. Changes in Body Weights

Individual body weight of each mouse was measured 1 day before the first CPA treatment, at first and second CPA treatment, at first/second and third/fourth test substance administration, and 12 h after the fourth (last) test substance administration (at sacrifice) using an automatic electronic balance (Precisa Instrument, Dietikon, Switzerland). To reduce the individual differences, the body weight gain during 3 days of CPA treatment, 2 days of test substance administration, and 5 days of the whole experimental period was also calculated as shown below.

*Body Weight Gain (g)*

1. Body weight gain during 3 days of CPA treatment = body weight at initial test substance administration − body weight at the day of first CPA treatment
2. Body weight gain during 2 days of test substance administration = body weight 12 h after the fourth test substance administration − body weight at initial test substance administration
3. Body weight gain during 5 days of whole experimental period = body weight 12 h after the fourth test substance administration − body weight at the day of first CPA treatment.

### 2.5. Lymphatic Organ Weight Measurement

At sacrifice, the weights of the thymus, spleen, and left submandibular LN were measured as absolute wet-weights individually and, to reduce the differences in individual body weights, the relative weights (% of body weights) were calculated using body weight at sacrifice and absolute weight by

(1) Relative  lymphatic  organ  weights %=Absolute  organ  weightBody  weight  at  sacrifice×100.

### 2.6. Hematology

About 200 *μ*L of whole blood sample was drawn from the posterior vena cava using a syringe with a 26-gauge needle under 2-3% isoflurane (Hana Pharm., Hwasung, Korea) inhalation anesthesia. The blood sample was collected into CBC bottles containing EDTA-2K (1.8 mg/mL of blood). All hematological measurements were conducted in Veterinary Teaching Hospital, College of Veterinary Medicine, Kyungpook National University (Daegu, Korea), using automated hematology cell counter (Cell-DYN3700, Abbott Laboratories, Abbott Park, IL, USA).

The 13 hematological items measured were as follows: total leukocyte numbers (WBC), differential counts (neutrophils, NEU; lymphocytes, LYM; monocytes, MONO; eosinophils, EOS; and basophils, BASO), erythrocyte numbers (RBC), hemoglobin concentrations (Hb), hematocrit (Hct), mean corpuscular volume (MCV), mean corpuscular hemoglobin (MCH), mean corpuscular hemoglobin concentration (MCHC), and platelet number (PLT).

### 2.7. Serum IFN-*γ* Level Measurement

For serum IFN-*γ* level measurement, about 0.5 mL of whole blood was collected from the vena cava at sacrifice under isoflurane inhalation anesthesia and centrifuged at 3,000 rpm for 10 min at 4°C to separate the serum. All serum samples were stored at −150°C in an ultradeepfreezer (Sanyo, Tokyo, Japan) until assay. Serum IFN-*γ* levels were calculated using mouse IFN-*γ* ELISA kit (BD Biosciences/Pharmingen, San Jose, CA, USA) according to the manufacturer's recommended protocols at pg/mL levels.

### 2.8. NK Cell Activity Measurement

Splenic and peritoneal NK cell activities were measured by a standard 51Cr release assay [29–31]. In brief, all mice were sacrificed and their splenocytes and peritoneal NK cells were collected. Spleen (10–20 mg) were separated and washed with RPMI-1640 medium (Gibco BRL, Grand Island, NY, USA) twice at 4°C and the homogenates were prepared. Peritoneal NK cells were collected by repeated intraperitoneal wash with RPMI medium. The splenic and peritoneal NK cells were mechanically disrupted by maceration through a wire mesh (Mesh Number 100, Sigma-Aldrich Co. LLC., St. Louise, MO, USA) wetted with RPMI-1640 medium. The mesh was washed with RPMI-1640 medium to collect as many cells as possible. The debris was allowed to settle, and the cell suspension was pelleted by centrifugation. RBCs were lysed by resuspending the pellet in cold 1% ammonium oxalate and incubating on ice for 10 min. The cells were pelleted and washed twice with Hanks Balanced Salt Solution (Gibco BRL, Grand Island, NY, USA). The peritoneal NK cells (1 × 10^5^ cells/mL to 2 × 10^5^ cells/mL) were cultured overnight in complete medium (Sigma-Aldrich, St. Louise, MO, USA). Splenocytes were cultured overnight in Dulbecco's modified Eagle medium (Invitrogen, Grand Island, NY, USA) in the absence or presence of recombinant IL-2 (1000 IU/mL; Proleukin Chiron, Emeryville, CA, USA). The HTLA-230 neuroblastoma target cells were labeled for 2 h with Na_2_^51^CrO_4_ (100 *μ*Ci/L × 10^6^ cells) (ICN Biomedicals, Asse, Belgium). Target cells were incubated for 6 h at 37°C with splenocytes or peritoneal macrophages as effector cells. The effector : target cell ratio was 100 : 1 for splenocytes and 10 : 1 for peritoneal cells. Supernatants were collected, and the amount of radioactivity released into the supernatants was counted with a gamma counter (Cobra 5002; Canberra Packard, Meriden, CT, USA). The percentage of specific target cell lysis was calculated using

(2) %  Specific   Cr51   release NK  cell  activity=Exp−SM−S100%.

Exp is the observed released ^51^Cr value, *S* is the spontaneously released ^51^Cr value, and *M* is the maximum released ^51^Cr value.

### 2.9. Splenic Cytokine Content Measurement

Splenic concentrations of TNF-*α*, IL-1*β*, and IL-10 were measured by ELISA using commercially available kits, mouse TNF-*α* ELISA kit (BD Biosciences/Pharmingen, San Jose, CA, USA), mouse IL-1*β* ELISA kit (Genzyme, Westborough, MA, USA), and mouse IL-10 ELISA kit (Genzyme, Westborough, MA, USA), as previously described [25, 31]. Approximately 10–15 mg of tissue samples were homogenized in a tissue grinder containing 1 mL of lysis buffer (PBS containing 2 mM PMSF and 1 mg/mL of aprotinin, leupeptin, and pepstatin A) as described by Clark et al. (1991) [32]. Analysis was performed with 100 mL of standard (diluted in lysis buffer) or 10, 50, or 100 mL of tissue homogenate. Each sample was run in duplicate, and a portion of the sample was analyzed for protein. Data are expressed as pg/mg of protein. For each assay, a standard curve was generated and, based on replicates of the measured absorbance, demonstrated an average coefficient of variance of <10%.

### 2.10. Histopathology

After weight measurement at sacrifice, some parts of the thymus, spleen, and left side of submandibular LN were separated and fixed in 10% neutral buffered formalin for at least 24 h. Then, paraffin-embedded 3 *μ*m-thick sections were prepared. Each slide was stained with hematoxylin and eosin for general histopathology. Histological evaluation was performed on the central zone of each organ, whenever possible. The histopathologist was blinded to group distribution during analysis. To observe the changes in detail, the total and cortex thicknesses of thymus (*μ*m/thymus), total thickness of central cross trimmed spleen (from apex of anterior border to centre of posterior border; mm/spleen), white pulp numbers (pulps/mm^2^ of spleen) and diameters (*μ*m/white pulps), total submandibular LN thicknesses (*μ*m/central regions), number of cortex lymphoid follicles (number/mm^2^ of cortex), and cortex thicknesses (*μ*m/LN) were also calculated using a computer-based automated image analyzer (i-Solution FL ver 9.1, IMT i-Solution Inc., Vancouver, Quebec, Canada) under a microscope (Nikon, Tokyo, Japan), according to previous report [25].

### 2.11. Statistical Analyses

All data were expressed as mean ± standard deviation (SD) of 10 mice. Multiple comparison tests for different dose groups were conducted. Variance homogeneity was examined using the Levene test [33]. If the Levene test indicated no significant deviations from variance homogeneity, the obtained data were analyzed by one-way ANOVA test, followed by least-significant differences multicomparison test to determine the pairs of group comparison that were significantly different. In case a significant deviation from variance homogeneity was observed in Levene test, a nonparametric comparison test, Kruskal-Wallis *H* test, was conducted. When a significant difference was observed in the Kruskal-Wallis *H* test, the Mann–Whitney *U* (MW) test was conducted to determine the specific pairs of group comparison that were significantly different. Statistical analyses were conducted using SPSS for Windows (Release 14.0K, IBM SPSS Inc., Armonk, NY, USA) [34]. In addition, the percent changes between intact vehicle and CPA control were calculated to observe the severity of myelosuppression induced by CPA treatment in this study, and the percent changes compared with CPA control and test substance-treated mice were calculated to understand the immunomodulatory effects of test materials using the following equations [35]:

(3) Percentage  changes  compared  with  intact  vehicle  control %=Data  of  CPA  control−Data  of  intact  control  mouseData  of  intact  control  mouse×100,

(4) Percentage  changes  compared  with  CPA  control %=Data  of  test  material  treated  mouse−Data  of  CPA  control  mouseData  of  CPA  control  mouse×100.

## 3. Results

### 3.1. Changes in Body Weight and Body Weight Gain

**Figure 2:**
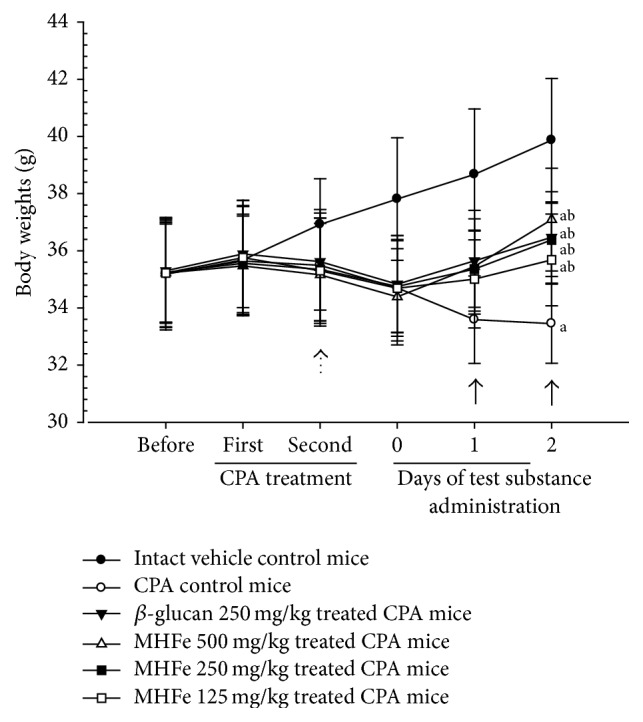
Body weights changes in intact or CPA-induced immunosuppressive mice. We selected ten mice per group showing lesser body weights as compared with intact vehicle control mice, and regarded animals at end of second CPA treatment as immunosuppressive; consequently, significant (*p* < 0.01) decreases of body weights were detected in all CPA-treated mice as compared with intact vehicle control mice from first/second administration day* (dot arrow)*. However, significant (*p* < 0.01 or *p* < 0.05) increases of body weights were demonstrated in *β*-glucan and MHFe 500 and 250 mg/kg treated mice from the day of third/fourth administration and at 12 hrs after last fourth administration in MHFe 125 mg/kg treated mice as compared with CPA control mice, respectively* (arrows)*. Values are expressed mean ± SD of eight mice; CPA: cyclophosphamide; MHFe: two-sweetener-excluded Kuseonwangdogo-based mixed herbal formula aqueous extracts. Before means 1 day before first CPA treatment; days 0 and 1 mean the day of first/second and third/fourth test substance administration, respectively; day 2 means the day of sacrifice, 12 hrs after last fourth test substance administration; ^a^*p* < 0.01 as compared with intact control mice by LSD test; ^b^*p* < 0.01 as compared with CPA control mice by LSD test.

We selected 10 mice per group with lower body weights than intact vehicle control mice and regarded as immunosuppressed animals at the end of second CPA treatment 34.67 ± 1.64 g of CPA-treated mice (32.1–37.8 g) and 37.81 ± 2.14 g of intact vehicle mice (34.5–41.7 g), respectively. A significant (*p* < 0.01) decrease in body weight was detected in all CPA-treated mice compared with intact vehicle control mice with significant (*p* < 0.01) decrease in body weight gain during 3 days of CPA treatment, 2 days of test substance administration, and 5 days of whole experimental period. However, a significant (*p* < 0.01 or *p* < 0.05) increase in body weight was observed in *β*-glucan treated and 250 and 500 mg/kg MHFe-treated mice from the day of third/fourth administration and at 12 h after the fourth (last) administration in 125 mg/kg MHFe-treated mice compared with CPA control mice. In addition, all test substance-treated mice showed a significant (*p* < 0.01) increase in body weight gain during the 2 days of test substance administration and 5 days of whole experimental period compared with control (Table 2, Figure 2).

### 3.2. Changes in Thymus Weight

A significant (*p* < 0.01) decrease in absolute and relative thymus weights were observed in CPA control mice compared with intact vehicle control. However, a significant (*p* < 0.01) increase in thymus weight was noticed in all test substance-administered mice, including 250 mg/kg *β*-glucan treated mice, compared with CPA control mice. In addition, all three dosages of MHFe dose-dependently inhibited CPA-induced thymic weight decrease, similar to *β*-glucan (Table 3).

### 3.3. Changes in Spleen Weight

A significant (*p* < 0.01) decrease in absolute and relative spleen weights was observed in CPA control mice compared with intact vehicle control. However, a significant (*p* < 0.01) increase in spleen weight was detected in all test substance-administered mice compared with CPA control mice. All three dosages of MHFe dose-dependently inhibited CPA-induced splenic weight decrease, similar to *β*-glucan (Table 3).

### 3.4. Changes in Submandibular Lymph Node Weight

A significant (*p* < 0.01) decrease in absolute and relative liver weights was observed in CPA control mice compared with intact vehicle control. A significant (*p* < 0.01 or *p* < 0.05) and dose-dependent increase in submandibular LN weight was observed in all MHFe-treated mice compared with CPA control mice. In addition, 250 mg/kg *β*-glucan significantly (*p* < 0.01) increased absolute and relative submandibular LN weights, similar to MHFe (Table 3).

### 3.5. Changes in Hematology

A significant (*p* < 0.01) decrease in WBC, RBC, Hb, Hct, and PLT without any meaningful change in differential WBC count, indicative of myelosuppressive nonregenerative anemia, panleukopenia, and thrombocytopenia, was observed in CPA control mice compared with intact vehicle control. However, these myelosuppressive changes in hematology were normalized by treatment with *β*-glucan. At the three dosages of MHFe, the mice showed evident dose-dependent inhibitory effects on CPA-induced myelosuppressive nonregenerative anemia, panleukopenia, and thrombocytopenia, similar to *β*-glucan-treated mice (Table 4).

### 3.6. Changes in Serum IFN-*γ* Levels

**Figure 3:**
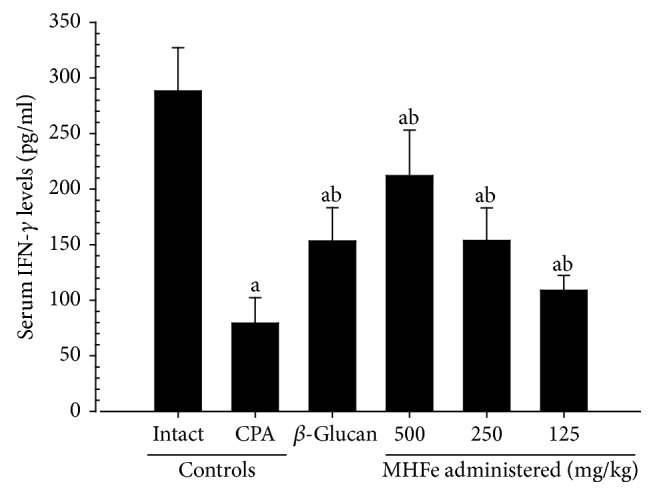
Serum IFN-*γ* levels in intact or CPA-induced immunosuppressive mice. Significant decreases of serum IFN-*γ* levels were noticed in CPA control mice as compared with intact vehicle control, respectively. All three different dosages of MHFe showed significant and clear dose-dependent increases of serum IFN-*γ* levels as compared with CPA control mice, respectively. In addition, *β*-glucan 250 mg/kg administered mice also showed increases of serum IFN-*γ* levels comparable to those of equal dosages of MHFe administered mice, in this study; values are expressed mean ± SD of eight mice; CPA: cyclophosphamide; MHFe: two-sweetener-excluded Kuseonwangdogo-based mixed herbal formula aqueous extracts; IFN: interferon; ^a^*p* < 0.01 as compared with intact control mice by LSD test; ^b^*p* < 0.01 as compared with CPA control mice by LSD test.

A significant (*p* < 0.01) decrease in serum IFN-*γ* levels was noticed in CPA control mice compared with intact vehicle control. All three dosages of MHFe significantly (*p* < 0.01 or *p* < 0.05) and dose-dependently increased serum IFN-*γ* levels compared with their levels in CPA control mice. In addition, 250 mg/kg *β*-glucan increased serum IFN-*γ* levels similar to MHFe (Figure 3).

### 3.7. Changes in NK Cell Activity

**Figure 4:**
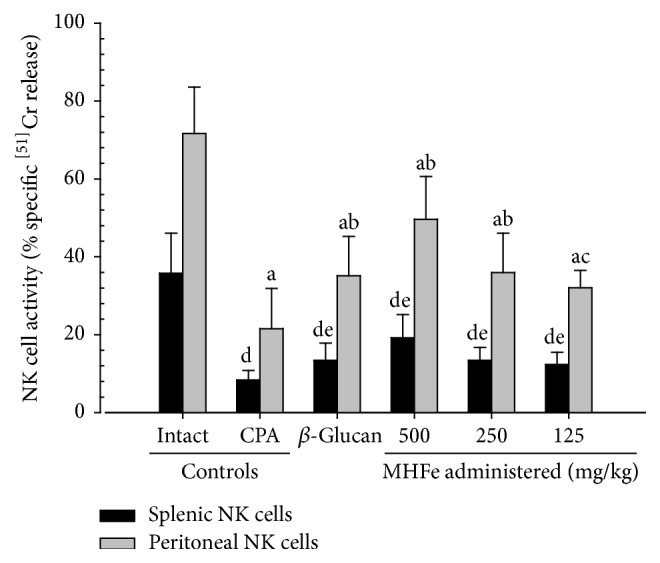
NK cell activities in intact or CPA-induced immunosuppressive mice. Significant decreases of splenic and peritoneal NK cell activities were observed in CPA control mice as compared with intact vehicle control mice, respectively. However, significant increases of splenic and peritoneal NK cell activities were detected in all test substance-administered mice including *β*-glucan 250 mg/kg oral administered mice as compared with CPA control mice, respectively. All three different dosages of MHFe-treated mice, especially, showed obvious dose-dependent inhibitory effects on CPA-induced decreases of NK cell activities as similar as equal dosages of *β*-glucan, in this experiment; values are expressed mean ± SD of eight mice; CPA: cyclophosphamide; MHFe: two-sweetener-excluded Kuseonwangdogo-based mixed herbal formula aqueous extracts; NK: natural killer; ^a^*p* < 0.01 as compared with intact control mice by LSD test; ^b^*p* < 0.01 and ^c^*p* < 0.05 as compared with CPA control mice by LSD test; ^d^*p* < 0.01 as compared with intact control mice by MW test; ^e^*p* < 0.01 as compared with CPA control mice by MW test.

A significant (*p* < 0.01) decrease in splenic and peritoneal NK cell activities were observed in CPA control mice compared with intact vehicle control mice. However, a significant (*p* < 0.01 or *p* < 0.05) increase in splenic and peritoneal NK cell activities was detected in all test substance-administered mice, including the reference drug 250 mg/kg *β*-glucan treated mice, compared with CPA control mice. All three dosages of MHFe exhibited a clear dose-dependent inhibitory effect on CPA-induced decrease in NK cell activities, similar to *β*-glucan (Figure 4).

### 3.8. Changes in Splenic Cytokine Content

A significant (*p* < 0.01) decrease in splenic TNF-*α*, IL-1*β*, and IL-10 content was observed in CPA control mice compared with intact vehicle control. However, a significant (*p* < 0.01 or *p* < 0.05) increase in splenic TNF-*α*, IL-1*β*, and IL-10 content was detected in all test substance-administered mice, including those given the lowest dosage (125 mg/kg) of MHFe, compared with CPA control mice. All three dosages of MHFe showed a clear dose-dependent inhibitory effect on CPA-induced decrease in splenic cytokines, similar to *β*-glucan (Table 5).

### 3.9. Effects on Lymphoid Organ Histopathology

**Figure 5:**
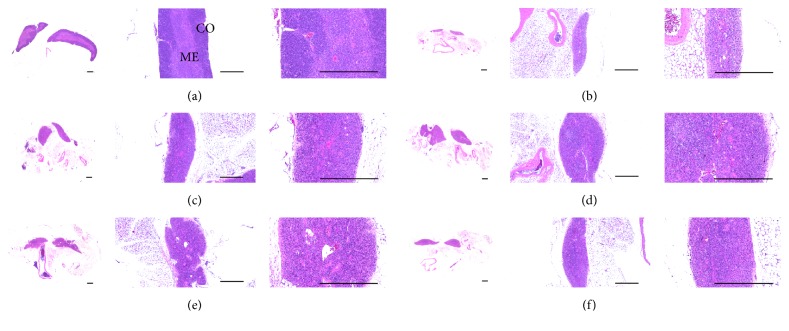
Representative histopathological images of the thymus, taken from intact or CPA-induced immunosuppressive mice. Noticeable atrophic changes related to the decrease of thymic cortex lymphoid cells were detected in CPA control mice as compared with intact vehicle control mice; consequently the thymus total and cortex thicknesses were significantly decreased in CPA control mice as compared with intact vehicle control mice, respectively. However, these thymic atrophic changes were significantly inhibited by treatment of *β*-glucan and all three different dosages of MHFe as compared with CPA control mice, respectively. All three different dosages of MHFe-treated mice, especially, showed obvious dose-dependent inhibitory effects on CPA-induced histopathological atrophic changes of the thymus similar to equal dosages of *β*-glucan, in this experiment; (a) distilled water-administered intact mice (vehicle control); (b) CPA-treated and distilled water-administered control mice (CPA control); (c) CPA-treated and *β*-glucan 250 mg/kg administered mice (*β*-glucan); (d) CPA-treated and MHFe 500 mg/kg administered mice (MHFe 500); (e) CPA-treated and MHFe 250 mg/kg administered mice (MHFe 250); (f) CPA-treated and MHFe 125 mg/kg administered mice (MHFe 125); CPA: cyclophosphamide; MHFe: two-sweetener-excluded Kuseonwangdogo-based mixed herbal formula aqueous extracts; CO: cortex; ME: medulla; all Hematoxylin-Eosin stain; scale bar: 400 *μ*m.

Atrophic changes related to the decrease in thymic cortex lymphoid cells were detected in CPA control mice compared with intact vehicle control mice. Consequently, the total thymus and cortex thicknesses significantly (*p* < 0.01) decreased in CPA control mice compared with intact vehicle control mice. However, these thymic atrophic changes were significantly (*p* < 0.01) inhibited by treatment with *β*-glucan and all three dosages of MHFe compared with CPA control mice. The three dosages of MHFe dose-dependently inhibited CPA-induced histopathological atrophic changes of the thymus, similar to *β*-glucan (Table 6, Figure 5).

### 3.10. Effects on Splenic Histopathology

**Figure 6:**
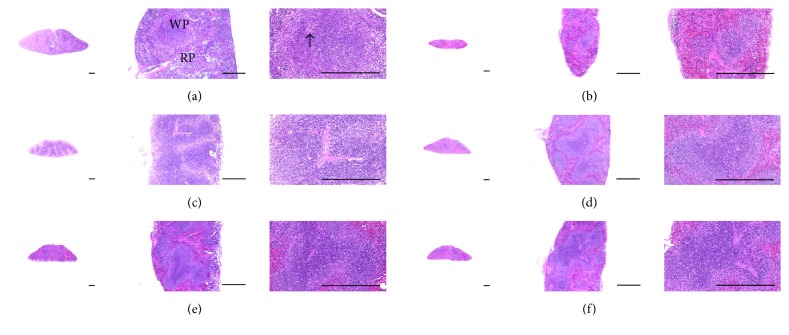
Representative histopathological images of the spleen, taken from intact or CPA-induced immunosuppressive mice. Obvious atrophic changes related to the decrease of splenic white pulp lymphoid cells were detected in CPA control mice as compared with intact vehicle control mice; consequently the total splenic thicknesses and white pulp numbers and diameters were significantly decreased in CPA control mice as compared with intact control mice, respectively. However, these splenic atrophic changes were markedly inhibited by treatment of *β*-glucan and MHFe 500, 250, and 125 mg/kg as compared with CPA control mice, respectively. All three different dosages of MHFe-treated mice, especially, showed clear and noticeable dose-dependent inhibitory effects on CPA-induced histopathological atrophic changes of the spleen similar to equal dosages of *β*-glucan, in this experiment; (a) distilled water-administered intact mice (vehicle control); (b) CPA-treated and distilled water-administered control mice (CPA control); (c) CPA-treated and *β*-glucan 250 mg/kg administered mice (*β*-glucan); (d) CPA-treated and MHFe 500 mg/kg administered mice (MHFe 500); (e) CPA-treated and MHFe 250 mg/kg administered mice (MHFe 250); (f) CPA-treated and MHFe 125 mg/kg administered mice (MHFe 125); CPA: cyclophosphamide; MHFe: two-sweetener-excluded Kuseonwangdogo-based mixed herbal formula aqueous extracts; WP: white pulp; RP: red pulps; arrow: central arteriole; all Hematoxylin-Eosin stain; scale bar: 400 *μ*m.

Atrophic changes related to the decrease in splenic white pulp lymphoid cells were detected in CPA control mice compared with intact vehicle control mice. The total spleen thickness, white pulp numbers, and diameters were significantly (*p* < 0.01) lower in CPA control mice than in intact control mice. However, these splenic atrophic changes were markedly inhibited by treatment with *β*-glucan and 125, 250, and 500 mg/kg MHFe compared with CPA control mice. All three dosages of MHFe showed noticeable dose-dependent inhibitory effect on CPA-induced histopathological atrophic changes in the spleen, similar to *β*-glucan (Table 6, Figure 6).

### 3.11. Effects on Submandibular Lymph Node Histopathology

**Figure 7:**
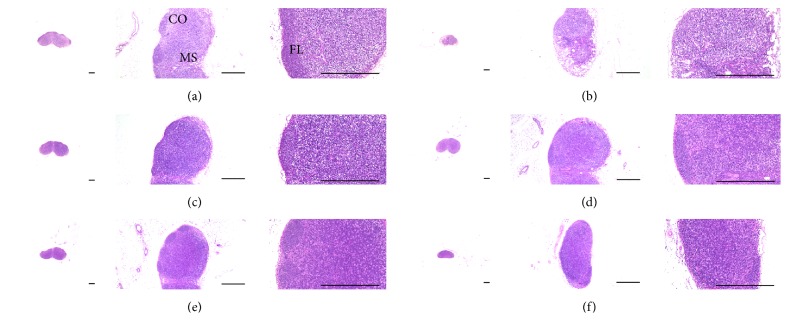
Representative histopathological images of the left submandibular LN, taken from intact or CPA-induced immunosuppressive mice. Diffused decreases of lymphoid cell-related atrophic changes were detected in the submandibular LN of CPA control mice as compared with intact control mice; consequently the total and cortex thicknesses and follicle numbers were significantly decreased in CPA control mice as compared with intact control mice, respectively. However, these submandibular lymph node atrophic changes were markedly and significantly inhibited by treatment of all test substances including *β*-glucan 250 mg/kg as compared with CPA control mice, and all three dosages of MHFe-treated mice, especially, showed clear dose-dependent inhibitory effects on CPA-induced histopathological atrophic changes of the submandibular LN similar to equal dosages of *β*-glucan, in this experiment; (a) distilled water-administered intact mice (vehicle control); (b) CPA-treated and distilled water-administered control mice (CPA control); (c) CPA-treated and *β*-glucan 250 mg/kg administered mice (*β*-glucan); (d) CPA-treated and MHFe 500 mg/kg administered mice (MHFe 500); (e) CPA-treated and MHFe 250 mg/kg administered mice (MHFe 250); (f) CPA-treated and MHFe 125 mg/kg administered mice (MHFe 125); CPA: cyclophosphamide; MHFe: two-sweetener-excluded Kuseonwangdogo-based mixed herbal formula aqueous extracts; LN: lymph node; CO: cortex; MS: medullary sinus; FL: follicle; all Hematoxylin-Eosin stain; scale bar: 400 *μ*m.

A decrease in lymphoid cell-related atrophic changes was detected in the submandibular LN of CPA control mice compared with intact control mice. The total and cortex thicknesses and follicle numbers were significantly (*p* < 0.01) lower in CPA control mice than in intact control mice. However, these submandibular LN atrophic changes were significantly (*p* < 0.01 or *p* < 0.05) inhibited by treatment with all test substances, including 250 mg/kg *β*-glucan, compared with CPA control mice. All three dosages of MHFe dose-dependently inhibited CPA-induced histopathological atrophic changes in the submandibular LN, similar to *β*-glucan (Table 6, Figure 7).

## 4. Discussion

Immune functions are indispensable as they constitute the host defenses against infections and therefore play a crucial role in maintaining health [36]. Decline in immune functions due to aging, chronic illnesses, physical and mental stress, or unhealthy lifestyles is a major clinical problem globally [36–38]. Drugs that normalize or modulate pathophysiological processes are known as immunomodulatory agents [39]. In recent years, there has been growing interest in the field of herbal medicine and search for promising compounds for identifying immunomodulatory compounds from natural products [11]. Herbal drugs enhance the natural resistance of the body against infection and their immunomodulatory activities have been reported in numerous plants [12, 13]. In this study, we observed the immunomodulatory effects of MHFe of two-sweetener-excluded Kuseonwangdogo, a traditional Korean immunomodulatory polyherbal prescription [14], on CPA-induced immunosuppressive mouse model. To induce immunosuppression in mice, CPA was intraperitoneally injected twice 3 days or 1 day before the initial test substance administration at 150 and 110 mg/kg. Test substances were orally administered 4 times 24 h after second CPA treatment at 12 h intervals, according to our previously established method [25]. *β*-glucan, a well-documented immunomodulatory polysaccharide [40], was used as the reference drug according to previous studies [25–28]. Twelve hours after the last (fourth) oral administration of the three dosages (125, 250, and 500 mg/kg) of MHFe or 250 mg/kg of *β*-glucan, changes in the body, thymus, spleen, and submandibular LN weights, 13 hematological parameters, serum IFN-*γ* levels, peritoneal and splenic NK cell activities, and splenic TNF-*α*, IL-1*β*, and IL-10 levels were monitored with histopathology of lymphoid organs. The total and cortex thicknesses of thymus, white pulp numbers, total and white pulp diameters of the spleen, lymphoid follicle numbers, and total and cortex thicknesses of submandibular LN were the histomorphometrical parameters evaluated in this study.

The dosage of *β*-glucan was selected as 250 mg/kg on the basis of previous* in vivo* efficacy tests [25–28]. The middle dosage of MHFe was selected as 250 mg/kg for direct comparison with *β*-glucan, and 500 and 125 mg/kg were selected as the highest and lowest dosages using common ratio 2, respectively.

Following CPA treatment in mice, a noticeable decrease in body weight and body weight gain, thymus, spleen, and submandibular LN weights, WBC, RBC, PLT, Hb, and Hct, serum IFN-*γ* levels, splenic TNF-*α*, IL-1*β*, and IL-10 content, and peritoneal and splenic NK cell activities was observed. In addition, depletion of lymphoid cells in thymic cortex, splenic white pulp, and submandibular LN-related atrophic changes of the lymphoid organs were observed, which suggested classic CPA-induced myelosuppression with nonregenerative anemia. However, these CPA-induced myelosuppression signs were dose-dependently inhibited by the oral administration of 125, 25, and 500 mg/kg MHFe compared with *β*-glucan. These results are direct evidence that MHFe has favorable immunomodulatory effects on CPA-induced myelosuppression and indicate that Kuseonwangdogo-based MHFe can be a potent immunomodulatory agent for treatment of various immune disorders.

Noticeable decrease in body weights has been observed after treatment with CPA by other investigators, indicating its direct toxicity [25, 41, 42], as well as decrease in lymphatic organ weight related to myelosuppressive depletion of peripheral lymphoid cells [11, 25, 43]. In this experiment, we also selected immunosuppressed animals based on lower body weight than intact vehicle control mice at the end of second CPA treatment. Consequently, a significant decrease in body weight was detected in all CPA-treated mice compared with intact vehicle control mice with significant decrease in body weight gain during 3 days of CPA treatment, 2 days of test substance treatment, and 5 days of whole experimental period. In addition, a significant decrease in thymus, spleen, and submandibular LN weights in CPA control mice compared with intact vehicle control mice was observed. However, a significant and dose-dependent increase in body, thymus, spleen, and submandibular LN weights were noticed with all three dosages of MHFe as well as with 250 mg/kg *β*-glucan compared with CPA control mice. In addition, 250 mg/kg MHFe-treated mice showed inhibitory effects on CPA-induced body and decrease in lymphatic organ weight, comparable to treatment with *β*-glucan. These findings constitute indirect evidence that MHFe modulated body immune responses. In general, the animals with enhanced immune system have showed relatively good growth patterns [44–46]. In this study, all intact vehicle control mice showed normal body weight increase in same age mice group [47, 48].

CPA is a widely used antineoplasic drug, employed alone or in combination with other products [49]. Used as an anticancer drug or in bone marrow transplantation conditioning regimes, CPA severely injures hematopoietic and lymphoid tissues, leading to a profound panleucopenia [23, 24, 50], with nonregenerative anemia and thrombocytopenia [23–25]. On observing the 13 items of hematology in this experiment, a significant decrease in WBC, RBC, Hb, Hct, and PLT without any meaningful change in differential WBC count, as indication of myelosuppressive nonregenerative anemia, panleukopenia, and thrombocytopenia, was also observed in CPA control mice compared with intact vehicle control. However, these myelosuppressive changes in hematology were normalized by treatment with *β*-glucan and all three dosages of MHFe. All three dosages of MHFe dose-dependently inhibited CPA-induced myelosuppressive nonregenerative anemia, panleukopenia, and thrombocytopenia, similar to *β*-glucan. These results constitute direct evidence that MHFe exhibits favorable and potent immunomodulatory effects through facilitation of myelohematopoiesis or inhibitory effects on CPA-induced myelosuppression.

The cytokine TNF-*α*, produced by various cell types, including splenocytes, was found to be associated with critical events leading to T-lineage commitment and differentiation [51]. TNF-*α* can enhance the* in vivo* immune response at doses much lower than those that cause weight loss or tissue toxicity. It enhances proliferation of B and T cells and promotes the generation of cytotoxic T cells. In addition, it enhances IL-2-induced immunoglobulin production and augments IL-2 stimulated natural killer cell activity and monocyte proliferation [52]. IL-1 is another cytokine released by various cell types, such as macrophages, dendritic cells, lymphocytes, endothelial cells, fibroblasts, and keratocytes, and two forms of IL-1, IL-1*α*, and IL-1*β*. They are both glycoproteins of 17 kDa; IL-1*β* is secreted by cells and IL-1*α* is membrane-bound. IL-1 is necessary for the successful initiation of some forms of immune response [53]. IL-10 is an immunosuppressive glycoprotein of 19–21 kDa secreted by Th2 cells, certain B cells, and activated macrophages. It is now clear that IL-10 primarily acts on activated macrophages to suppress their secretion of IL-1, IL-12, TNF-*α*, and reactive oxygen radicals [52]. IFN-*γ* is a glycoprotein of 20–25 kDa produced by CD8+ T cells, Th1 cells, and NK cells in response to IL-2. It has a complex effect on B and T cell functions and enhances the NK cell and macrophage activities [52]. CPA depletes the immune T cells and reduces cytokine release in various immune cells [25, 43, 54]. A marked decrease in stimulatory cytokines, splenic TNF-*α*, IL-1*β*, and IL-10 content, and blood IFN-*γ* levels may be due to decline in circulating immune cells after CPA treatment. However, a significant increase in splenic TNF-*α*, IL-1*β*, and IL-10 was observed with all dosages of test substance administered, including the lowest dosage of 125 mg/kg MHFe, which suggested the potent immunomodulatory effect of MHFe compared with *β*-glucan.

Under CPA-induced myelosuppression, marked functional disorders of various immune cells, including NK cells, have been observed [55–57]. The activation of these immune cells was highlighted as a new treatment regimen for cancer [58–60]. In this study, marked decrease in both splenic and peritoneal NK cell activities was also noticed after CPA treatment, but significant dose-dependent increase in splenic and peritoneal NK cell activities was observed in all MHFe-treated mice similar to *β*-glucan-treated mice. This suggests that the potent immunomodulatory effects of MHFe may be mediated through the activation of NK cells, at least in part.

Histopathologically, atrophic changes related to depletion of lymphoid cells, particularly T cell subsets, are induced in the thymus, LN, and spleen after treatment with CPA [25, 42], which was also observed in this experiment. Atrophic changes related to decrease in thymic cortex, splenic white pulp, and submandibular LN lymphoid cells were detected in CPA control mice. This was confirmed by histomorphometrical analysis in this study. However, these lymphatic organ-related atrophic changes were significantly and dose-dependently inhibited at all dosages of MHFe, comparable to *β*-glucan treatment in this experiment. Once again, these findings are direct evidence that MHFe exhibits favorable and potent immunomodulatory effects through facilitation of myelohematopoiesis or inhibitory effects on CPA-induced myelosuppression.

## 5. Conclusion

MHFe significantly inhibited CPA-induced myelosuppression, a direct evidence of immunomodulatory effects through myelohematopoiesis, compared with *β*-glucan (reference drug). Therefore, Kuseonwangdogo-based MHFe is a promising novel potent immunomodulatory agent that can be used in the treatment of various immune disorders with less toxicity.

## Figures and Tables

**Table 1:** Composition of MHFe used in this study.

Herbs	Scientific names	Amounts (g)
Nelumbinis Semen	*Nelumbo nucifera *Gaertn.	870
Dioscoreae Rhizoma	*Dioscorea batatas *Decaisne.	870
Hoelen Alba	*Poria cocos *Wolf	870
Coicis Semen	*Coix lacryma-jobi *L. var. *mayuen *Stapf.	870
Hordei Fructus Germinatus	*Hordeum vulgare *L.	435
Lablab Semen Album	*Dolichos lablab *L.	435
Euryales Semen	*Euryale ferox *Salisb.	435

Total	7 types	4785

MHFe: two-sweetener-excluded Kuseonwangdogo-based mixed herbal formula aqueous extracts.

**Table 2:** Body weight gains in intact or CPA-induced immunosuppressive mice.

Groups	Periods					
	Body weights at:			Body weight gains during:		
	First CPA treatment [A]	First/second administration of test substance [B]	At sacrifice [C]	CPA treatment (3 days) [B-A]	Test substance administration (3 days) [C-B]	Whole experimental periods (5 days) [C-A]
Controls						
Intact	35.68 ± 1.90	37.81 ± 2.14	39.87 ± 2.16	2.13 ± 0.94	2.06 ± 0.44	4.19 ± 1.14
CPA	35.64 ± 1.91	34.70 ± 1.69^a^	33.45 ± 1.38^a^	−0.94 ± 0.81^a^	−1.25 ± 0.75^c^	−2.19 ± 0.89^a^
*β*-Glucan	35.89 ± 1.88	34.83 ± 1.70^a^	36.47 ± 1.60^ab^	−1.06 ± 0.59^a^	1.64 ± 0.35^de^	0.58 ± 0.67^ab^
MHFe						
500 mg/kg	35.47 ± 1.75	34.39 ± 1.68^a^	37.09 ± 1.80^ab^	−1.08 ± 0.35^a^	2.70 ± 0.24^ce^	1.62 ± 0.32^ab^
250 mg/kg	35.56 ± 1.72	34.75 ± 1.60^a^	36.38 ± 1.28^ab^	−0.81 ± 0.63^a^	1.63 ± 0.79^e^	0.82 ± 0.87^ab^
125 mg/kg	35.76 ± 2.00	34.69 ± 1.84^a^	35.68 ± 1.60^ab^	−1.07 ± 0.61^a^	0.99 ± 0.68^ce^	−0.08 ± 0.78^ab^

Values are expressed mean ± SD of eight mice, g; CPA: cyclophosphamide; MHFe: two-sweetener-excluded Kuseonwangdogo-based mixed herbal formula aqueous extracts; ^a^*p* < 0.01 as compared with intact control mice by LSD test; ^b^*p* < 0.01 as compared with CPA control mice by LSD test; ^c^*p* < 0.01 and ^d^*p* < 0.05 as compared with intact control mice by MW test; ^e^*p* < 0.01 as compared with CPA control mice by MW test.

**Table 3:** Organ weights in intact or CPA-induced immunosuppressive mice.

Groups	Organs					
	Absolute weights (g)			Relative weights (% of body weights)		
	Thymus	Spleen	LN	Thymus	Spleen	LN
Controls						
Intact	0.068 ± 0.012	0.115 ± 0.019	0.021 ± 0.006	0.171 ± 0.034	0.288 ± 0.053	0.052 ± 0.012
CPA	0.016 ± 0.003^d^	0.046 ± 0.010^d^	0.005 ± 0.002^d^	0.047 ± 0.010^d^	0.136 ± 0.029^d^	0.014 ± 0.007^a^
*β*-Glucan	0.028 ± 0.006^de^	0.067 ± 0.010^de^	0.010 ± 0.003^de^	0.077 ± 0.015^de^	0.183 ± 0.027^de^	0.027 ± 0.009^ab^
MHFe						
500 mg/kg	0.035 ± 0.009^de^	0.079 ± 0.014^de^	0.012 ± 0.003^de^	0.095 ± 0.027^de^	0.213 ± 0.037^de^	0.038 ± 0.008^ab^
250 mg/kg	0.027 ± 0.008^de^	0.066 ± 0.010^de^	0.010 ± 0.002^de^	0.074 ± 0.021^de^	0.182 ± 0.026^de^	0.026 ± 0.006^ab^
125 mg/kg	0.022 ± 0.004^de^	0.061 ± 0.009^de^	0.008 ± 0.002^de^	0.061 ± 0.011^de^	0.171 ± 0.021^de^	0.023 ± 0.006^ac^

Values are expressed mean ± SD of eight mice; CPA: cyclophosphamide; MHFe: two-sweetener-excluded Kuseonwangdogo-based mixed herbal formula aqueous extracts; LN: submandibular lymph node, left side; ^a^*p* < 0.01 as compared with intact control mice by LSD test; ^b^*p* < 0.01 and ^c^*p* < 0.05 compared with CPA control mice by LSD test; ^d^*p* < 0.01 as compared with intact control mice by MW test; ^e^*p* < 0.01 as compared with CPA control mice by MW test.

**Table 4:** Hematological values in intact or CPA-induced immunosuppressive mice.

Items	Groups					
	Controls			MHFe		
	Intact	CPA	*β*-glucan	500 mg/kg	250 mg/kg	125 mg/kg
WBC (K/*μ*l)	7.51 ± 1.73	0.30 ± 0.11^c^	0.85 ± 0.17^cd^	1.24 ± 0.64^cd^	0.83 ± 0.16^cd^	0.63 ± 0.12^cd^
Differential count						
LYM%	74.83 ± 9.73	74.80 ± 6.83	74.41 ± 10.72	73.51 ± 10.40	74.91 ± 7.40	74.61 ± 9.74
NEU%	18.40 ± 8.88	18.97 ± 6.96	18.84 ± 10.89	20.02 ± 10.80	18.88 ± 7.42	19.01 ± 10.04
MONO%	4.73 ± 1.80	4.66 ± 1.24	4.52 ± 1.68	4.64 ± 2.03	4.64 ± 1.72	4.68 ± 1.40
EOS%	0.50 ± 0.70	0.49 ± 0.44	0.47 ± 0.46	0.50 ± 0.52	0.48 ± 0.42	0.50 ± 0.54
BASO%	0.54 ± 0.54	0.55 ± 0.55	0.56 ± 0.49	0.55 ± 0.47	0.54 ± 0.46	0.54 ± 0.36
RBC (M/*μ*l)	9.16 ± 1.17	5.91 ± 1.03^c^	7.21 ± 0.38^cd^	7.79 ± 0.36^cd^	7.30 ± 0.52^cd^	7.01 ± 0.42^cd^
Hb (g/dl)	18.87 ± 1.50	13.63 ± 1.84^c^	16.62 ± 0.57^cd^	17.30 ± 0.79^cd^	16.64 ± 0.84^cd^	16.01 ± 0.91^cd^
Hct (%)	47.43 ± 1.62	40.62 ± 0.46^c^	42.02 ± 0.77^cd^	43.22 ± 0.85^cd^	42.04 ± 0.48^cd^	41.64 ± 0.89^cd^
MCV (fl)	51.96 ± 1.84	51.76 ± 3.05	52.08 ± 2.36	51.79 ± 1.87	52.08 ± 2.81	52.20 ± 2.88
MCH (pg)	18.01 ± 1.05	17.93 ± 0.95	18.03 ± 1.24	17.86 ± 1.53	18.17 ± 1.83	17.89 ± 1.48
MCHC (g/dl)	19.28 ± 1.14	19.30 ± 1.69	19.40 ± 1.62	19.20 ± 1.26	19.38 ± 1.72	19.28 ± 1.06
PLT (M/*μ*l)	1501.00 ± 115.25	965.70 ± 116.63^a^	1162.90 ± 86.95^ab^	1259.80 ± 121.90^ab^	1157.30 ± 129.42^ab^	1113.00 ± 106.78^ab^

Values are expressed mean ± SD of eight mice. Full name of hematological items were listed in Table 2; CPA: cyclophosphamide. MHFe: two-sweetener-excluded Kuseonwangdogo-based mixed herbal formula aqueous extracts; WBC: white blood cell, LYM: lymphocytes, NEU: neutrophils, MONO: monocytes, EOS: eosinophils, BASO: basophils, RBC: red blood cell, Hb: hemoglobin concentration, Hct: hematocrit, MCV: mean corpuscular volume, MCH: mean corpuscular hemoglobin, MCHC: mean corpuscular hemoglobin concentration, and PLT: platelet count; ^a^*p* < 0.01 as compared with intact control mice by LSD test; ^b^*p* < 0.01 as compared with CPA control mice by LSD test; ^c^*p* < 0.01 as compared with intact control mice by MW test; ^d^*p* < 0.01 as compared with CPA control mice by MW test.

**Table 5:** Changes in the splenic cytokine contents in intact or CPA-induced immunosuppressive mice.

Groups	Cytokines		
	Splenic cytokine contents (pg/ml)		
	Tumor necrosis factor-*α*	Interleukin-1*β*	Interleukin-10
Controls			
Intact	117.28 ± 24.00	67.90 ± 13.36	96.56 ± 24.33
CPA	39.42 ± 15.85^a^	20.75 ± 10.49^a^	34.47 ± 14.55^a^
*β*-Glucan	66.03 ± 18.46^ab^	37.55 ± 11.83^ab^	61.73 ± 10.77^ab^
MHFe			
500 mg/kg	89.77 ± 15.49^ab^	50.49 ± 13.55^ab^	74.23 ± 12.51^ab^
250 mg/kg	67.25 ± 13.71^ab^	38.59 ± 12.40^ab^	61.08 ± 14.06^ab^
125 mg/kg	57.09 ± 11.12^ac^	33.87 ± 9.11^ac^	53.84 ± 13.69^ac^

Values are expressed mean ± SD of eight mice, pg/ml; CPA: cyclophosphamide; MHFe: two-sweetener-excluded Kuseonwangdogo-based mixed herbal formula aqueous extracts; ^a^*p* < 0.01 as compared with intact control mice by LSD test; ^b^*p* < 0.01 and ^c^*p* < 0.05 compared with CPA control mice by LSD test.

**Table 6:** Histomorphometrical analysis in intact or CPA-induced immunosuppressive mice.

Items	Groups					
	Controls			MHFe		
	Intact	CPA	*β*-glucan	500 mg/kg	250 mg/kg	125 mg/kg
Thymus-thickness						
Total (*μ*m)	756.66 ± 152.51	221.18 ± 61.77^a^	464.30 ± 82.11^a^	650.03 ± 113.44^bc^	465.12 ± 103.29^ac^	353.65 ± 108.21^ac^
Cortex (*μ*m)	291.95 ± 90.11	50.23 ± 17.40^a^	132.10 ± 38.87^ac^	196.10 ± 30.33^ac^	133.30 ± 26.84^ac^	106.36 ± 19.50^ac^
Spleen-thickness						
Total (*μ*m)	1882.93 ± 211.98	773.40 ± 173.96^a^	1220.70 ± 113.88^ac^	1320.15 ± 155.10^ac^	1216.14 ± 98.56^ac^	1063.07 ± 150.82^ac^
Cortex (*μ*m)	413.46 ± 55.59	185.92 ± 47.26^e^	298.46 ± 22.99^eg^	350.14 ± 55.58^fg^	304.31 ± 20.94^eg^	260.68 ± 34.28^eg^
WP number (/mm^2^)	15.60 ± 3.10	4.70 ± 1.57^e^	9.20 ± 1.48^eg^	13.70 ± 2.00^g^	9.40 ± 1.51^eg^	7.90 ± 1.10^eg^
LN-thickness						
Total (*μ*m)	1198.93 ± 126.22	472.08 ± 112.35^a^	861.22 ± 135.38^ac^	994.86 ± 128.55^ac^	891.35 ± 131.31^ac^	615.36 ± 80.32^ad^
Cortex (*μ*m)	867.66 ± 106.04	286.07 ± 104.07^a^	594.14 ± 145.05^ac^	737.86 ± 130.89^bc^	671.25 ± 123.16^ac^	320.09 ± 97.73^ad^
Follicle number (/mm^2^)	12.70 ± 2.41	2.70 ± 1.95^a^	8.90 ± 1.29^ac^	10.50 ± 1.58^ac^	9.00 ± 1.70^ac^	4.90 ± 1.20^ac^

Values are expressed mean ± SD of eight mice; CPA: cyclophosphamide; MHFe: two-sweetener-excluded Kuseonwangdogo-based mixed herbal formula aqueous extracts; WP: White pulp; LN: submandibular lymph node, left sides; ^a^*p* < 0.01 and ^b^*p* < 0.05 as compared with intact control mice by LSD test; ^c^*p* < 0.01 and ^d^*p* < 0.05 as compared with CPA control mice by LSD test; ^e^*p* < 0.01 and ^f^*p* < 0.05 as compared with intact control mice by MW test; ^g^*p* < 0.01 as compared with CPA control mice by MW test.
